# Plasma 25-Hydroxy Vitamin D is not Associated with Acne Vulgaris

**DOI:** 10.3390/nu10101525

**Published:** 2018-10-17

**Authors:** Abdullah Al-Taiar, Mona AlKhabbaz, Abdur Rahman, Reem Al-Sabah, Lemia Shaban, Saeed Akhtar

**Affiliations:** 1 Department Community Medicine and Behavioural Sciences, Faculty of Medicine, Kuwait University, Box 24923, Safat 13110, Kuwait; reem1@hsc.edu.kw (R.A.-S.); saeed.akhtar@hsc.edu.kw (S.A.); 2 Quality and Accreditation Directorate, Ministry of Health, Box 5, State 13001, Kuwait; mona.alkhabbaz@hsc.edu.kw; 3 Department of Food Science and Nutrition; College of Life Sciences, Kuwait University, Box 5969, Safat 13060, Kuwait; abdurrahman.ahmad@ku.edu.kw (A.R.); dr.lemiashaban@gmail.com (L.S.)

**Keywords:** Acne vulgaris, vitamin D, Kuwait

## Abstract

Few studies have investigated the association between Acne vulgaris (AV) and vitamin D level. In this study we aimed to investigate the association between 25-hydroxyvitamin D (25-OH-VitD) level and AV in a country with plenty of sunshine. A cross-sectional study was conducted on 714 adolescents who were randomly selected from public schools using multistage cluster random sampling with probability proportional to size. 25-OH-VitD levels were measured using liquid chromatography-tandem mass spectrometry (LC-MS/MS). The Global Acne Grading System (GAGS) was used to assess the severity of AV. Data on potential confounders were collected from the parents through a self-administered questionnaire, and from the adolescents using a face-to-face interview. Of 714 participants, 351 (41.16%) were males. The mean (standard deviation (SD)) age was 12.28 (0.81) years. AV was observed in 479 (67.1%) adolescents. There was no significant association between 25-OH-VitD level and clinically assessed AV before or after adjusting for potential confounders. This was consistent whether 25-OH-VitD was fitted as a continuous variable or categorized using acceptable cutoff points or tertiles. In this study vitamin D status was not associated with AV, therefore our data do not support vitamin D supplementation either to treat or to prevent AV.

## 1. Introduction

### Background

It is estimated that 9.4% of the world’s population is affected by acne vulgaris (AV) with the highest prevalence among adolescents [1]. It is the most common skin disease globally [2,3] and ranked as the eighth most common disease worldwide [4,5]. Although isolated skin diseases are unlikely to be fatal conditions, during 2013 skin diseases were the fourth leading cause of disability worldwide [6]. Among skin diseases, AV was ranked the second leading cause of disability-adjusted life years after dermatitis (atopic, contact, and seborrheic dermatitis) [6]. In 2013, The American Academy of Dermatology reported lost productivity due to AV for patients and caregivers that was comparable to the productivity cost of skin cancer [7]. Additionally, AV puts a significant burden to healthcare services. In the United States, AV is the fourth most common reason for seeking medical consultation among adolescents [2]. AV may negatively impact quality of life and lead to social anxiety and isolation [8,9]. It may reduce self-confidence as well as academic performance [10]. AV has also been linked to depression, and in most severe forms suicidal thoughts and suicide attempts [11,12,13,14].

The link between vitamin D level and AV has attracted attention recently with anecdotal evidence from few case-control studies [15,16,17,18]. Recently, low levels of vitamin D have been noticed in various skin disease conditions [19]. Furthermore, studies have identified vitamin D receptors in sebocytes [20,21], the cells lining sebaceous glands, which suggests a role of vitamin D in the acne pathophysiology. Only a few epidemiological studies have investigated the association between a low vitamin D level and AV [15,16,17,18]. The statistical analysis in these studies was merely a crude comparison of the mean vitamin D level between cases and controls without taking into account confounding by other factors, the distribution of vitamin D levels in the study groups, or matching the cases to the controls [15,17]. We have previously demonstrated a high prevalence of vitamin D deficiency among adolescents in Kuwait [22]; and if shown to be related to AV or its severity, this will provide a rationale for conducting randomized controlled trials to test the efficacy of vitamin D supplementation on treatment or prevention of AV. Therefore, this analytical cross-section study aimed to investigate the association between 25-hydroxyvitamin D (25-OH-VitD) level and AV among adolescents in Kuwait, a country with plenty of sunshine.

## 2. Method

Kuwait is a small country at latitude (29.3759° N) with a population of 4.2 million, of whom about 21% are in the age range of 10–19 years (public authority for civil information 2017). Schools’ enrolment is high in Kuwait for both males and females. The study population included male and female students from public middle/high schools, typically aged 11–16 years.

The participants in this study were previously recruited in a cross-sectional study aimed to study vitamin D status in Kuwait (Project No. WF02/13) [22]. A list of all public schools with the number of students in each school was obtained from The Ministry of Education. This was stratified by gender and governorate and then was used to select a random sample of students through multistage cluster random sampling. We selected one school for boys and one school for girls from each governorate using a probability proportional to size sampling method, which takes into account the number of students in each school. We distributed the sample between different governorates based on the total number of students in that governorate relative to the total number of students in the country (proportionate allocation). After obtaining written informed consent from the parents and verbal assent from the adolescents, data were collected from the parents using a self-administered questionnaire, while data from the adolescents were obtained through a face-to-face interview by trained dedicated personnel using a structured questionnaire. The questionnaire was developed after extensive literature review and was pilot tested on 20 students who were not included in the study.

Blood samples (5 mL) were collected in gel-containing tubes (SST II advance, BD vacutainer) from each child by a trained nurse; and the samples were protected from light. On the same day, the samples were centrifuged at 2000× *g* for 15 min and the serum was stored at −80 °C until laboratory analysis. We measured plasma 25-OH-VitD level, which is the best marker of vitamin D status. Plasma 25-OH-VitD is the major circulating form of vitamin D that reflects both vitamin D produced in the skin after exposure to sunlight and vitamin D intake from foods [23]. 25-OH-VitD was measured by liquid chromatography-tandem mass spectrometry (LC-MS/MS)—a recommended method for vitamin D assessment in research studies [24]. We measured the serum intact parathyroid hormone (PTH) using the Access Intact PTH chemiluminescent immunoassay with the UnicelDxI 800 Beckman Coulter analyzer using commercial kit (Cat. # A16972).

In an independent study with new parental consent, data on AV and associated factors were collected using a face-to-face interview and clinical examination from the study group. We assessed the presence or absence of AV in addition to the severity of AV using Global Acne Grading Scale (GAGS). Based on GAGS, the face, chest, and back are divided into six areas (forehead, each cheek, nose, chin, chest, and back) with a factor assigned to each area according to the surface area and the density of pilosebaceous units. Depending on severity, each type of lesion is given a value: zero for no lesion; one for comedones; two for papules; three for pustules; and four for nodules. The local score for each area is calculated as Factor × Grade (0–4). Local scores are added to generate the global score, and acne severity is classified based on the global score (1–18 = mild; 19–30 = moderate; 31–38 = severe; and >39 = very severe). In a subgroup of 186 participants, the diagnosis of AV and grading was conducted by two independent physicians and the degree of agreement was calculated (Kappa 60.7% for presence or absence of acne). The Ethics Committee at The Ministry of Health in Kuwait approved this study (No. 2015/248). 

Data were entered and linked to the previously collected data using a unique identifier, and analyzed using the Statistical Package for Social Sciences (SPSS) version 24 and Stata version 12. We used logistic regression to investigate the association between 25-OH-VitD and AV status. We separately analyzed self-reported AV and AV diagnosed by clinical examination as outcome variables. Vitamin D status was categorized using the acceptable cutoff points [25,26] as vitamin D deficiency (<50 nmol/L), vitamin D insufficiency (50 to less than 75 nmol/L), and vitamin D sufficiency (≥75 nmol/L). Furthermore, severe vitamin D deficiency was defined as <25.0 nmol/L [27]. Potential confounders were classified into three groups; i. socio-demographic factors (age, gender, nationality, governorate, education of the father, education of the mother and total number of siblings); ii. taking supplement during the last six months. BMI categories were calculated based on measured weight and height and was classified according to WHO growth charts, total time spent on physical activity (gathered using a questionnaire that has been validated using accelerometers (Spearman correlation 0.92; *p* < 0.001 for total steps count; not published); and iii. consumption of selected food items, which included frequency of consumption (every day, 4–6 days/week, 1–3 days/week, one day/week, never) of each fruits, vegetable, chips and fried food, whole cream milk, and low-fat milk during the last three months. First, crude association was evaluated and then each group of confounders was introduced to the model sequentially. Because AV was a common outcome, we used a modified version of logistic regression that calculates the prevalence ratio instead of odds ratio as described by Zhang and Yu [28]. Because of ongoing debate on the above mentioned cutoff points of vitamin D, [29,30,31,32], we repeated the analysis treating 25-OH-VitD level as a continuous variable and after categorizing it into tertiles.

## 3. Results

Of the 1416 participants from whom we took blood samples, 714 participants were examined for AV. Table 1 shows the socio-demographic characteristics of 1416 participants in the original study and 714 participants who were examined for AV. Overall, those who participated in the current study were not substantially different from those who participated in the original study. The mean (SD) age was 12.28 (0.81) years and the majority were Kuwaiti nationals. 

The prevalence of self-reported AV was 320 (44.8%), which was significantly higher among females than males (56.2% vs. 33.0%; respectively *p* < 0.001). The prevalence of AV diagnosed by clinical examination was 479 (67.1%) and was also significantly higher among females compared to males (74.1% vs. 59.8; respectively *p* < 0.001). Based on GAGS, 379 participants (53.08% of all study groups) had mild AV, while 100 participants (14.01% of all study group) had moderate AV, and no participants had severe AV.

25-OH-VitD levels were not significantly (*p* = 0.07) different between those with (median 30.0; IQR 20.9–44.1 nmol/L) and without (median 32.5; IQR 22.5-47.7 nmol/L) AV diagnosed by clinical examination. However, 25-OH-VitD levels were significantly (*p* = 0.002) lower (median 27.15; IQR 18.7–42.75 nmol/L) among those with self-reported AV than those without it (median 33.0; IQR 22.5–47.0 nmol/L). Of 714 students, 131 (18.35%) were on vitamin supplement in the last six months. AV diagnosed by clinical examination was not significantly different between those who took a supplement and those who did not (72.52% vs. 65.87%, respectively; *p* = 0.143). Furthermore, PTH was not significantly (*p* = 0.238) different between those with (median 6.02; IQR 4.52–8.27 pmol/L) and without (median 5.86; IQR 4.10–8.05 pmol/L) AV diagnosed by clinical examination.

There was no evidence for an association between vitamin D status and AV diagnosed by clinical examination before or after adjusting for potential confounders (Table 2). Furthermore, both in crude and adjusted analysis, vitamin D level, either as a continuous or categorical as tertiles, was associated with AV diagnosed by clinical examination (data not shown). In contrast, there was significant association between vitamin D status and self-reported AV in univariable analysis but this association did not hold after adjusting for potential confounders (Table 3). We also repeated this analysis once treating vitamin D as a continuous variable and second after categorizing vitamin D levels into tertiles. Both analyses showed no association between vitamin D and self-reported AV before and after adjusting for potential confounders (data not shown). 

## 4. Discussion

This study aimed to investigate the association between vitamin D level and AV among adolescents in a country with abundant sunshine. To the best of our knowledge this is the largest study that addressed this issue. There was no association between vitamin D status and clinically assessed AV before or after adjusting for potential confounders (Table 2). However, there was an association between self-reported AV and vitamin D status only in univariate analysis but not in multivariate analysis (Table 3).

Previously only a few studies have investigated the association between vitamin D and AV [15,16,17,18]. The first was in Iran and included 35 patients with AV and 40 healthy controls, and showed no difference in the medians levels of vitamin D between cases of AV and controls [16]. In this study, there was no multivariate analysis to adjust for confounders and patients with AV due to polycystic ovary syndrome were included in the study groups (12 among cases and 3 among controls). The second study was in Turkey, where patients with nodulocystic AV (*n* = 43) had significantly lower mean levels of vitamin D than the subjects in the control group (*n* = 46) [15]. The analysis was merely a crude comparison between cases and controls without adjusting for potential confounders. The third study was in South Korea; and reported significantly higher prevalence of vitamin D deficiency (<12 ng/mL) in AV patients (*n* = 80) compared to healthy controls (*n* = 80) (48.8% vs. 22.5%; *p* = 0.019) [17] without adjusting for potential confounders or accounting for matching cases to controls by age and gender. In this study, however, the authors randomized 39 cases of AV who had a vitamin D deficiency to either 1000 IU/day vitamin D supplement (*n* = 20) or placebo (*n* = 19) and reported improvement of AV severity in the treatment group. This trial, however, was small and the analysis did not go beyond univariate analysis. In a recent cases-control study, authors reported significantly higher prevalence of vitamin D deficiency (<50 nmol/L) among AV cases compared to controls without adjusting for confounders [18]. As mentioned earlier, the evidence based on the results of these studies is no more than anecdotal and therefore, further studies are needed to investigate the association between vitamin D and AV. 

It is hypothesized that the impact of vitamin D on AV is due to the possible anti-inflammatory effect. The presence of *Propionibacterium acnes* in AV lesion leads to secretion of various inflammatory cytokines, including IL-8 and IL-12 in addition to the recruitment of activated T helper 1 (Th1) and T helper 17 (Th17) lymphocytes to the site of early AV lesions [33]. Vitamin D modulates both the innate and adaptive immune responses by governing lymphocyte activation, survival, differentiation, and maturation in addition to inhibition of the expression of interferon and pro-inflammatory cytokines in monocytes (IL-1, IL-6, TNF-α, IL-8 and IL-12) [34]. Vitamin D also inhibits T-cell proliferation and suppresses the transcription of genes encoding Th1, which activates cell-mediated immunity, suppresses the production of B-cell opsonizing antibodies, and reduces Th17 (providing anti-microbial immunity) [35]. As such, the association between vitamin D and AV is presumed to exist only in patients with AV inflammatory lesions. In the above mentioned study [17], authors reported association between vitamin D level and AV severity in patients with inflammatory lesions but no association was found in patients with non-inflammatory forms of AV. We repeated our analysis after excluding adolescents with non-inflammatory lesions and found no association between vitamin D and AV. Finally, it has been suggested that adolescents with severe AV may suffer from severe psychological stress and tend to avoid spending extended periods outdoors, suggesting a potential explanation for the association between vitamin D and AV [17]. 

While the lack of association between vitamin D level and AV in our study is likely to be genuine, it can be attributed to several reasons. First, our adolescents were uniformly vitamin D deficient or insufficient (only 31 adolescents were vitamin D sufficient according to the proposed cutoff points). Such common exposure will make epidemiological studies like ours inefficient in detecting association between vitamin D and AV even if it exists. Second, it is possible that those with severe AV were absent from school during the data collection and if they had lower vitamin D level, this would attenuate the association between vitamin D and AV (loss to follow-up bias). Of note all AV cases in our study were either moderate or mild with no severe cases of AV—probably because we have young study group, which is, as mentioned above, likely to attenuate the association between vitamin D level and AV, if it exists. We compared vitamin D level between those who participated in this study and those who did not and found small but statistically significant difference, median (IQ) 30.9 (21.3–44.9) nmol/L and 27.85 (17.6–43.1) nmol/L, respectively; *p* = 0.005).

This is the first large scale study that investigated the association between vitamin D status and AV. 25-OH-VitD was measured using LC-MS/MS, which is the recommended method of vitamin D measurement in research studies [25]. Nevertheless, the study has several limitations including the fact that we only managed to examine about half of the original study participants for AV. Although there is no strong evidence from literature that AV is associated with school absenteeism, one could speculate that those severely affected by AV were missed because they are more likely to be absent from the schools hence less likely to participate in the study. This is likely to attenuate the association between vitamin D and AV. Finally, we measured 25-OH-VitD only at one point in time, which might have not reflected the long-term profile of vitamin D status. This is also likely to attenuate the association between 25-OH-D and AV, if it exists (non-differential misclassification).

In conclusion, we found no association between vitamin D status and AV in this study. Although our data do not support vitamin D supplementation either to treat or to prevent AV, it is important to improve vitamin D level among adolescents for several other health benefits. Further prospective studies to investigate the relationship between vitamin D level and AV are warranted.

## Figures and Tables

**Table 1:** Socio-demographic characteristics of 1416 participants in the original study and 714 participants in the current study.

Characteristics	Participants with Blood Samples		Participants Examined for Acne Vulgaris	
Age, Mean (SD) years	12.48	(0.94)	12.28	(0.81)
	*n*	(%)		
Gender				
Male	694	(49.01)	351	(49.16)
Nationality				
Kuwaiti	1081	(76.34)	511	(71.57)
Non-Kuwait	335	(23.66)	203	(28.43)
Father’s Education ^1^				
No formal education	15	(1.08)	6	(0.86)
Primary/Intermediate	221	(15.98)	85	(12.14)
Secondary (high school)	344	(24.87)	174	(24.86)
Diploma	261	(18.87)	139	(19.86)
University & above	542	(39.19)	296	(42.29)
Mother’s Education ^2^				
No formal education	31	(2.22)	12	(1.70)
Primary/Intermediate	152	(10.89)	58	(8.23)
Secondary (high school)	304	(21.78)	153	(21.70)
Diploma	304	(21.78)	150	(21.28(
University & above	605	(43.34)	332	)47.09(
Father’s Income ^3^ (Kuwaiti Dinars)				
Less than 500	91	(6.64)	54	(7.81)
500 to 1000	304	(22.19)	160	(23.15)
1001 to 1500	421	(30.73)	210	(30.39)
1501 to 2000	219	(15.99)	102	(14.76)
More than 2000	173	(12.63)	94	(13.60)
Do not wish to tell	162	(11.82)	71	(10.27)
Mother’s Employment Status ^4^				
Housewife	488	(35.16)	225	(32.01)
Paid employment	680	(48.99)	361	(51.35)
Others	220	(15.85)	117	(16.64)
Housing ^5^				
Rented flat	510	(36.51)	281	(39.91)
Rented house	163	(11.67)	79	(11.22)
Owned flat	59	(4.22)	41	(5.82)
Owned house	665	(47.60)	303	(43.04)

^1^ Missing for 33 participants in original study and 14 participants in the current study; ^2^ Missing for 20 participants in original study and 9 participants in the current study; ^3^ Missing for 46 participants in original study and 23 in the current study; ^4^ Missing for 28 participants in the original study and 11 participants in the current study; ^5^ Missing for 19 participants in the original study and 10 participants in the current study. SD: standard deviation.

**Table 2:** Association between vitamin D and acne vulgaris (clinical exam) before and after adjusting for potential confounders.

Vitamin D Status	Model 1 PR [95%CI]	Model 2 PR [95%CI]	Model 3 PR [95%CI]	Model 4 PR [95%CI]
Severe deficiency (*n* = 247) 25-OH-VitD levels <25.0 nmol/L	1.00 [Reference]	1.00 [Reference]	1.00 [Reference]	1.00 [Reference]
Deficiency (*n* = 328) 25-OH-VitD levels <50 nmol/L	0.94 [0.82–1.05]	1.02 [0.88–1.14]	1.02 [0.87–1.14]	1.00 [0.85–1.13]
Insufficiency (*n* = 108) 25-OH-VitD levels 50–75 nmol/L	0.92 [0.75–1.07]	1.07 [0.87–1.22]	1.08 [0.88–1.22]	1.06 [0.84–1.22]
Sufficiency (*n* = 31) 25-OH-VitD levels ≥75 nmol/L	0.87 [0.59–1.11]	0.99 [0.67–1.22]	1.00 [0.68–1.24]	0.96 [0.62–1.22]
*p*-value	0.061	0.88	0.86	0.89

PR: Prevalence ratio; CI: confidence intervals; 25-OH-VitD: 25-hydroxyvitamin D; Model 1: unadjusted; Model 2: adjusted for socio-demographic factors (age, gender, nationality, governorate, education of the father, education of the mother and total number of siblings); Model 3: adjusted for variables in the Model 2 in addition to taking supplement during the last six months, BMI categories, total time spent on physical activity; Model 4: adjusted for variables in Model 3 in addition to consumption of selected food items.

**Table 3:** Association between vitamin D and acne vulgaris (self-reported) before and after adjusting for potential confounders.

Vitamin D Status	Model 1 PR [95%CI]	Model 2 PR [95%CI]	Model 3 PR [95%CI]	Model 4 PR [95%CI]
Severe deficiency (*n* = 247) 25-OH-VitD levels <25.0 nmol/L	1.00 [Reference]	1.00 [Reference]	1.00 [Reference]	1.00 [Reference]
Deficiency (*n* = 328) 25-OH-VitD levels <50 nmol/L	0.81 [0.65–0.98]	1.03 [0.82–1.24]	1.02 [0.81–1.22]	1.01 [0.80–1.23]
Insufficiency (*n* = 108) 25-OH-VitD levels 50-75 nmol/L	0.68 [0.49–0.91]	1.10 [0.80–1.40]	1.09 [0.79–1.39]	1.02 [0.72–1.34]
Sufficiency (*n* = 31) 25-OH-VitD levels ≥75 nmol/L	0.94 [0.58–1.33]	1.33 [0.88–1.71]	1.33 [0.87–1.71]	1.24 [0.76–1.66]
*p*-value	0.03	0.52	0.54	0.79

PR: Prevalence ratio; 25-OH-VitD: 25-hydroxyvitamin D; Model 1: unadjusted; Model 2: adjusted for socio-demographic factors (age, gender, nationality, governorate, education of the father, education of the mother and total number of siblings); Model 3: adjusted for variables in the Model 2 in addition to taking supplement during the last six months, BMI categories, total time spent on physical activity; Model 4: adjusted for variables in Model 3 in addition to consumption of selected food items.
